# Childbirth attitudes in parous and nulliparous women: the roles of cognitive flexibility, perceived social support, and obstetric factors

**DOI:** 10.1186/s40359-026-04864-0

**Published:** 2026-05-25

**Authors:** İlknur Kiraz Avcı, Selma Mahmutoglu, Mehmet Avcı

**Affiliations:** 1 Psychiatry Clinic, Rize State Hospital, Rize, Turkey; 2 Gynecology and Obstetrics Clinic, Rize State Hospital, Rize, Turkey; 3 Guidance and Psychological Counseling Program, Recep Tayyip Erdogan University, Rize, 53200 Turkey

**Keywords:** Childbirth attitudes, Social support, Cognitive flexibility, Türkiye, Pregnancy

## Abstract

**Background:**

Childbirth is frequently associated with increased negative emotions, leading to psychological challenges for many pregnant women. In this study, childbirth attitudes are defined as encompassing both fear of childbirth and negative evaluative attitudes toward childbirth. Factors shaping these attitudes, including perceived social support and cognitive flexibility in parous and nulliparous women, remain underexplored. This study aims to examine the influence of perceived social support and cognitive flexibility on childbirth attitudes among parous and nulliparous women based on sociodemographic and obstetric factors.

**Methods:**

A cross-sectional research design included 362 (55.5% parous) pregnant women in Turkey. The data were analyzed using Pearson correlation, ANOVA, hierarchical regression, and network analysis.

**Results:**

Hierarchical regression analyses revealed that cognitive flexibility showed the strongest association among the examined variables with childbirth attitudes among both parous and nulliparous women (R² = 0.067). The dimensions of cognitive flexibility, namely, control and alternatives were found to account for unique variance and were the only significant associated factors of childbirth fear (CFI-Control: β = -0.460, *p* < 0.001; CFI-Alternatives: β = 0.128, *p* = 0.024). In network analyses, CFI-Alternatives exhibited the highest strength, betweenness, and closeness in the nulliparous group, whereas friends and family were most central in the parous group.

**Conclusions:**

These findings suggest that cognitive flexibility is modestly associated with childbirth attitudes, particularly within Turkish culture, where social support may not consistently mitigate fear. The study emphasizes the importance of targeting cognitive flexibility in prenatal care, particularly for pregnant women without prior childbirth experience.

## Background

Pregnancy and childbirth—pivotal milestones in a woman’s life—are essential not only for the continuation of the human species, but also for the establishment of familial bonds and the broader social fabric. This period is widely recognized as a major life transition, often experienced as a psychosocially demanding phase requiring substantial adjustment and role reorganization [1]. The complex process of pregnancy and childbirth is shaped by a combination of individual attitudes, beliefs, and environmental factors, all of which play a crucial role in determining the trajectory of women’s adaptation during this critical period. These interacting factors may either facilitate adaptive coping and personal growth or, conversely, hinder adjustment and increase difficulty in managing the transition [2].

### Childbirth attitudes

Birth attitudes are broadly defined as beliefs, perceptions, and emotional responses that individuals hold regarding the childbirth process [3, 4]. These attitudes can significantly affect how women perceive, prepare for, and cope with the birth experience, shaping their overall approach to this critical life event [5]. Previous research documented a range of psychological, cultural, and social factors that affect childbirth attitudes. For instance, prenatal experiences, familial beliefs about childbirth, and community norms were reported to contribute to shaping women’s perceptions of the childbirth process [6, 7]. Furthermore, numerous studies also highlighted the impact of previous personal birth experiences, media portrayals, and family perspectives on birth attitudes, emphasizing the crucial role that these factors play in shaping women’s expectations from and understanding of childbirth [8, 9]. Moreover, evidence suggests that childbirth attitudes—operationalized in this study as reflecting both fear of childbirth and negative cognitive-evaluative perceptions—are strongly shaped by individuals’ beliefs and personality traits [10–12].

Considering the central role that birth attitudes play in influencing women’s perception of the childbirth process, these attitudes exert a profound impact on postpartum outcomes, including mother-infant attachment and the subsequent risk of developing postpartum depression [13–15]. In this study, more favorable childbirth attitudes are reflected by lower CAQ scores, indicating reduced fear of childbirth and more adaptive cognitive–emotional evaluations of the birth experience. Women with negative attitudes towards childbirth frequently report lower satisfaction, which can impact their postpartum emotional health. In previous research, this dissatisfaction was linked to a higher likelihood of postpartum depression and anxiety [16, 17]. Moreover, women reporting negative childbirth experiences are less likely to desire future pregnancies and may prefer cesarean sections in subsequent births. For instance, Ghanbari-Homayi et al. [18] documented that negative childbirth attitudes correlated with reduced willingness for future pregnancies and a marked preference against vaginal deliveries, suggesting a lasting impact on reproductive decisions. Therefore, considering the significant emotional and psychological impact of childbirth attitudes on women’s wellbeing, it is essential to explore the factors that can support women in managing these challenges.

### Cognitive flexibility

Cognitive flexibility, defined as individuals’ ability to adapt to new situations and challenges [19, 20], is a dynamic process encompassing acceptance, rather than avoidance, of unpleasant internal experiences while engaging in behaviors aligned with personal values. This involves mindfully persisting in values-driven actions despite the presence of distressing thoughts, emotions, or physical sensations [21]. In the literature, cognitive flexibility appears to be linked to reduced psychological distress, with recent studies highlighting similar patterns within the perinatal population. For instance, a recent study highlighted that women exhibiting higher levels of psychological flexibility reported fewer symptoms of depression and anxiety [22]. Although available research on cognitive flexibility among pregnant women is limited, several relevant studies linked inflexibility to postpartum depression [21] or anxiety disorders in the immediate postpartum period [23]. In addition, there are some indications that cognitive flexibility among pregnant women may be limited [24], which may hinder women’s ability to cope with the challenges of pregnancy, because cognitive flexibility plays a key role in managing fear during the birthing process and promoting positive changes in birth attitudes [25].

### Social support

To date, extensive evidence is available showing that the provision of robust social support for the expectant mother is a key strategy to prevent or mitigate pregnancy complications and adverse birth outcomes associated with mental illness [26–28]. Broadly defined by the extent to which social relationships fulfill specific needs (e.g., emotional, instrumental, affectionate, and tangible support) or facilitate social integration [29, 30], social support helps to alleviate negative birth attitudes, leading to a more positive perspective on childbirth [31, 32]. Importantly, the nature of social support can considerably vary across different cultural contexts, affecting how pregnant women perceive childbirth. In cultures where family bonds are emphasized (e.g., collectivistic cultures), women may report higher satisfaction with their birth experiences and more positive attitudes towards the process [33]. In Turkey, childbirth is primarily considered a woman’s responsibility, which can limit men’s involvement in the process. However, available research indicates that many women desire spousal support during delivery, indicating a shift towards recognizing the importance of shared experiences in childbirth [34]. This shift highlights how evolving attitudes towards family support can be associated with more positive women’s perceptions of childbirth and greater emotional preparedness and overall birth experience.

### The present study

As discussed in the introduction, understanding the interaction among key variables that contribute to the development of a positive and resilient mindset—namely, childbirth attitudes, social support, and cognitive flexibility—is of critical importance. Although previous studies have examined these variables either individually or in limited combinations [11, 16, 18, 25], research addressing their interrelationships within a comprehensive and integrated framework remains scarce. Moreover, existing studies often treat pregnant women as a homogeneous group [28, 35–37], without adequately accounting for differences related to prior birth experience. However, parity is likely to shape women’s psychological processes, expectations, and coping mechanisms during pregnancy [29]. Addressing this distinction constitutes a key contribution of the present study, as the psychological profile of women experiencing childbirth for the first time (nulliparous) may differ substantially from that of women with previous obstetric experience (parous), particularly in terms of sources of social support and the level of cognitive flexibility required to manage childbirth-related fears and expectations.

From a theoretical perspective, cognitive flexibility may facilitate adaptive reappraisal of childbirth-related experiences, thereby reducing fear responses, whereas perceived social support may operate as an external coping resource that buffers stress; however, the relative influence of these mechanisms is expected to vary according to parity, with nulliparous women relying more on cognitive appraisal processes and parous women drawing more on prior experiential and social resources. Accordingly, examining the pattern and strength of associations among these variables within each group is essential to better understand how cognitive flexibility, perceived social support, and childbirth attitudes are structurally interrelated rather than operating in isolation. The present study seeks to address these critical gaps in the existing literature. Accordingly, the primary objectives are as follows: (1) to examine whether childbirth attitudes, perceived social support, and cognitive flexibility differ between parous and nulliparous women, taking sociodemographic and obstetric characteristics into account; (2) to determine the extent to which perceived social support and cognitive flexibility are associated with childbirth attitudes during pregnancy; and (3) to identify the underlying pattern of interrelations among childbirth attitudes, perceived social support, and cognitive flexibility, and their subcomponents within each group.

## Methods

### Study design and participants

A cross-sectional research design was used. With regard to the study participants, we recruited a convenience sample of pregnant women referred to the Gynecology and Obstetrics Department of Rize State Hospital, Turkey, between November 15 and December 31, 2024. The research team conducted standardized face-to-face interviews to gather questionnaire data at the clinic. Before beginning the survey, all participants signed an informed consent form, ensuring that they understood the study and its purposes. All participants were assured of anonymity and were allowed to withdraw from the study at any time. The approval for the study was obtained from the Committee of the Ministry of Health, Rize Provincial Health Directorate (No: E-64960800-799-259434310/2024).

A total of 388 women were initially recruited for the study. The participant selection process was conducted in two distinct stages to ensure data integrity. In the first stage, an obstetrician performed a clinical screening based on the pre-defined inclusion criteria: (1) aged 18 years or older; (2) carrying a singleton pregnancy; (3) being classified as low-risk for obstetric complications; and (4) having no history of or current psychiatric treatment. Only individuals meeting all these clinical parameters were invited to participate. In the second stage, following the administration of the survey instruments, an exclusion criterion was applied to address data quality; 26 participants were excluded due to incomplete or missing responses in the questionnaires. No additional missing data were present in the remaining included cases. The excluded questionnaires were removed solely due to incomplete responses, and the pattern of missingness did not indicate any systematic structure across the study variables. Moreover, the excluded participants did not differ meaningfully from the final sample in available demographic characteristics, suggesting that the use of complete-case analysis is unlikely to have introduced significant bias. Consequently, the final analysis was conducted with a dataset comprising 362 pregnant women, providing a robust sample for the study’s objectives. A power analysis was conducted using G*Power (version 3.1.9.7) to determine whether the sample size was adequate for the planned analysis. Assuming a medium effect size (f² = 0.15), an alpha level of 0.05, a statistical power of 0.95, and 5 predictors, the minimum required sample size was calculated as 138 participants. Accordingly, the sample size of the present study exceeded the required minimum, indicating that the study had sufficient statistical power. The mean age of the sample was 29.92 years (SD = 4.25). About half of the participants (49%) were in their third trimester of pregnancy (see Table 1 for further details).

**Table 1:** Sociodemographic characteristics of participants (parous, nulliparous, and total sample)

	Parous		Nulliparous		Total			
	*N*	%	*N*	%	*N*	%	Test statistic	*p*
Education								
Literate	8	4.0	3	1.9	11	3.00	X^2^=26.105	< 0.001**
Primary School	25	12.4	2	1.2	27	7.50		
High School	83	41.3	53	32.9	136	37.60		
University	85	42.3	103	64.0	188	51.90		
Economic Status								
Low	3	1.5	7	4.3	10	2.80	4.423^f^	0.099
Medium	194	96.5	147	91.3	341	94.20		
High	4	2.0	7	4.3	11	3.00		
Family type								
Nuclear	176	87.6	152	94.4	328	90.60	X^2^=4.926	0.026*
Extended	25	12.4	9	5.6	34	9.40		
Week of Pregnancy								
1–12 weeks	47	23.4	36	22.4	83	22.90	X^2^=0.773	0.680
13–24 weeks	54	26.9	50	31.1	104	28.70		
Over 24 weeks	100	49.8	75	46.6	175	48.30		
Pregnancy Type								
Normal	159	79.1	150	93.2	309	85.40	X^2^=14.147	< 0.001**
IVF	42	20.9	11	6.8	53	14.60		
Health problem								
Yes	26	12.9	14	8.7	40	11.0	X^2^=1.635	0.201
No	175	87.1	147	91.3	322	89.0		
Number of Births (Parous only)								
1	135	67.2	0	0.0	135	37.30		
2	48	23.9	0	0.0	48	13.30	462.023^f^	< 0.001**
3	14	7.0	0	0.0	14	3.90		
4	4	2.0	0	0.0	4	1.10		
Previous type of birth (Parous only)								
Vaginal birth	89	44.3	0	0.0	89	24.30	X^2^=357.976	< 0.001**
Cesarean	112	55.7	0	0.0	112	30.90		
Abortion / stillbirth								
Yes	55	27.4	21	13.0	76	21.00	X^2^=11.051	< 0.001**
No	146	72.6	140	87.0	286	79.00		
Information source								
Doctor	117	58.2	82	50.9	199	55.00	X^2^=37.709	< 0.001**
Midwife/Nurse	59	29.4	19	11.8	78	21.50		
Relatives/Friend	15	7.5	30	18.6	45	12.40		
TV/social media	10	5.0	30	18.6	40	11.00		
Books/Magazines	0	0.0	0	0.0	0	0.00		

*p < 0.05, ***p < 0.001, f: Fisher Exact test, X^2^: Chi-square test

### Measures

#### Sociodemographic form

To collect the study participants’ demographic information, we developed the sociodemographic form to gather comprehensive data on the study participants’ personal and obstetric characteristics. Along with basic sociodemographic information, such as age, education level, employment status, and number of children, the form also included specific details related to the participants’ pregnancy and maternity experience. These included the previous birth status (e.g., first-time or multiple births), the type of pregnancy (e.g., singleton or multiple), and the current gestational week.

### Childbirth Attitudes Questionnaire (CAQ)

Childbirth attitudes were measured using the CAQ developed by Lowe [35]. The validity of the Turkish version of this scale was established by Dönmez et al. [38]. The scale comprises 16 items rated on a four-point Likert-type scale (1 = no anxiety, 4 = high anxiety), with total scores ranging from 16 to 64. Higher scores on this instrument are associated with more pronounced negative attitudes toward childbirth. The Cronbach’s α for CAQ in the validation study was 0.82; in the present study, it amounted to 0.90.

#### Multidimensional Scale of Perceived Social Support (MSPSS)

The Multidimensional Scale of Perceived Social Support (MSPSS), developed by Zimet et al. [39], was employed to assess perceived social support from family, friends, and significant others. The instrument consists of 12 items rated on a 7-point Likert-type scale, ranging from 1 (very strongly disagree) to 7 (very strongly agree). Total scores on the scale range from 12 to 84, with higher scores indicating a greater level of perceived social support. The MSPSS was adapted into Turkish by Eker and Arkar [40], who reported an internal consistency of 0.87 for the total score. In the present study, the Cronbach’s alpha coefficient was calculated as 0.85.

#### Cognitive Flexibility Inventory (CFI)

The CFI [41] was employed to evaluate women’s ability to generate alternative, adaptive, appropriate, and balanced thoughts in challenging situations. This 20-item measure consists of two subscales: 13 items assess the ability to perceive potential alternatives in life situations and human behaviors, while further 7 items evaluate the tendency to perceive challenging situations as controllable. Each item was rated on a 5-point scale, ranging from 1 (not appropriate at all) to 5 (completely appropriate). Higher scores on the scale indicated a greater level of cognitive flexibility. The Turkish adaptation of the scale was conducted by Gülüm and Dağ [42], who reported an internal consistency coefficient of 0.90. In the present study, Cronbach’s alpha coefficient for the total scale amounted to 0.81, indicating good internal consistency in the current study.

### Data analysis

SPSS v27 statistical package program and JASP 0.19.1.0 were used to complete all statistics. Skewness-kurtosis values were examined to determine the distribution of the data. Skewness–kurtosis values being in the range of -2, + 2 indicate that scores were normally distributed [43, 44]. In addition, visual control by histograms and Q-Q plots indicated that the data were normally distributed. When the assumption of normality was met, the comparison of two independent groups was made using the Independent Samples t-test, while when the assumption was not met, the Mann-Whitney U test was used. When the assumption of normality was met for the comparison of three or more independent groups, the ANOVA test was applied, and when it was not met, the Kruskal-Wallis test was run. Bonferroni post-hoc analyses were conducted to identify the specific group or groups causing the difference. The network structure was estimated using 5,000 bootstrap resamples, and centrality indices (betweenness, closeness, and strength) were computed and reported for each variable. Edge weights were interpreted as pairwise associations between variables, with higher absolute values indicating stronger relationships. The Network analyses were conducted using JASP (version 0.19.1.0), and parameter estimates were evaluated within a 95% confidence interval framework to assess the precision of the obtained network statistics.

## Results

### Demographic and obstetric characteristics of participants

Table 1 presents the distribution of sociodemographic characteristics of the participants. More than half of the participants (51.9%) had a university education, and the majority (94.2%) reported a medium economic level. The distribution of participants according to family type indicated that 90.6% had a nuclear family, while 9.4% had an extended family. When examining the distribution of participants based on the week of pregnancy, 22.9% were in the first trimester, 28.7% were in the second trimester, and 48.3% were in the third trimester. Overall, 55.5% of the participants were parous, whereas 44.5% were nulliparous. The sociodemographic and obstetric variables, along with comparisons between parous and nulliparous individuals, are presented in Table 1.

The relationship between participants’ sociodemographic characteristics and their previous birth status was analyzed using the chi-square test (Table 1). According to the assumption of the chi-square test, when more than 20% of the cells have an expected count of less than 5, Fisher’s exact chi-square test was used. Significant associations were observed between previous birth status and education level, family type, pregnancy type, type of previous birth, and history of abortion/stillbirth (*p* < 0.001). In contrast, no statistically significant relationship was detected between previous birth and economic status, pregnancy week, or health problems (*p* > 0.05).

### Mean differences across MSPSS, CAQ, CFI, and correlation analyses

Table 2 presents descriptive statistics for the study measures. The MSPSS total score was 70.74 ± 14.35, with subscale means of 25.67 ± 4.39 for family support, 23.25 ± 5.82 for friend support, and 21.82 ± 7.04 for significant other support. The CAQ mean score was 32.4 ± 10.24. For the CFI, the total mean score was 71.36 ± 13.35, with subscale means of 23.62 ± 5.23 for control and 47.74 ± 10.31 for alternatives.

**Table 2:** Descriptive statistics and correlations among study variables

MSPSS-Family	Mean$\:\pm\:\:$SD	Cronbach’s Alpha	MSPSS Family	MSPSS Friend	MSPSS Significant other	MSPSS Total	CAQ	CFI Control	CFI Alternatives	CFI Total
	25.67 ± 4.39	0.882	1	0.525^**^	0.489^**^	0.651^**^	-0.048	0.138^**^	0.149^**^	0.195^**^
MSPSS-Friend	23.25 ± 5.82	0.892	0.610^**^	1	0.531^**^	0.853^**^	− 0.126^*^	0.113^*^	0.148^**^	0.159^**^
MSPSS-Significant other	21.82 ± 7.04	0.882	0.457^**^	0.531^**^	1	0.846^**^	-0.102	0.056	0.077	0.082
MSPSS Total	70.74 ± 14.35	0.908	0.778^**^	0.853^**^	0.846^**^	1	− 0.115^*^	0.109^*^	0.136^**^	0.147^**^
CAQ	32.4 ± 10.24	0.908	-0.045	− 0.126^*^	-0.102	− 0.115^*^	1	− 0.191^**^	0.016	-0.062
CFI Control	23.62 ± 5.23	0.933	0.117^*^	0.113^*^	0.056	0.109^*^	− 0.191^**^	1	0.413^**^	0.711^**^
CFI Alternatives	47.74 ± 10.31	0.786	0.122^*^	0.148^**^	0.077	0.136^**^	0.016	0.413^**^	1	0.934^**^
CFI Total	71.36 ± 13.35	0.913	0.140^**^	0.159^**^	0.082	0.147^**^	-0.062	0.711^**^	0.934^**^	1

*SD* Standard deviation, *MSPSS* Multidimensional Scale of Perceived Social Support, *CAQ* Childbirth Attitudes Questionnaire, *CFI* Cognitive Flexibility Inventory, Pearson correlation coefficients are reported for all variables except MSPSS-Family, for which Spearman correlation was used due to non-normal distribution

Note. *N* = 362, *p < 0.05, **p < 0.01

The relationships between the scales and their subdimensions were examined using Pearson correlation analysis for normally distributed variables and Spearman correlation analysis for non-normally distributed variables. In general, perceived social support was positively associated with cognitive flexibility and negatively associated with negative childbirth attitudes, although the effect sizes were small. MSPSS–Family was weakly positively correlated with CFI Control (*r* = 0.138, *p* < 0.01), CFI Alternatives (*r* = 0.149, *p* < 0.01), and overall CFI (*r* = 0.195, *p* < 0.001), indicating that higher levels of family support are associated with greater cognitive flexibility. MSPSS–Friend was weakly negatively correlated with CAQ (*r* = -0.126, *p* = 0.017), suggesting that higher peer support is associated with lower levels of negative childbirth attitudes, and was weakly positively correlated with CFI Control (*r* = 0.113, *p* = 0.031), CFI Alternatives (*r* = 0.148, *p* = 0.005), and overall CFI (*r* = 0.159, *p* = 0.002). Similarly, total MSPSS scores were weakly negatively correlated with CAQ (*r* = -0.115, *p* = 0.029) and positively correlated with CFI Control (*r* = 0.109, *p* = 0.038), CFI Alternatives (*r* = 0.136, *p* = 0.010), and overall CFI (*r* = 0.147, *p* = 0.005), suggesting modest associations between perceived social support and cognitive functioning. Finally, CAQ was weakly negatively correlated with CFI Control (*r* = -0.191, *p* < 0.001), indicating that higher levels of negative childbirth attitudes were associated with lower perceived cognitive control (Table 2).

### Comparison of parous and nulliparous women regarding MSPSS, CFI, and CAQ

The results showed that there was no statistically significant difference between parous and nulliparous women regarding MSPSS-Family, MSPSS-Friend, MSPSS-Significant Other, and overall MSPSS scores (*p* > 0.05) (Table 3). However, a statistically significant difference was observed in the CAQ scores (t=-4.936, *p* < 0.001), with nulliparous (35.28 ± 10.01) scoring significantly higher compared to parous women (30.1 ± 9.85). In terms of cognitive flexibility, parous women scored significantly higher on the CFI Control subscale (t = 3.397, *p* < 0.001), the CFI Alternatives subscale (t = 6.675, *p* < 0.001), and the overall CFI score (t = 6.529, *p* < 0.001). Parous women (75.24 ± 9.86) had higher overall cognitive flexibility scores than nulliparous women (66.52 ± 15.43). Effect sizes were calculated to complement statistical significance and indicated negligible differences in perceived social support between parous and nulliparous women (MSPSS-Friend: d = -0.05; MSPSS-Significant Other: d = − 0.26; MSPSS-Family: *r* = 0.04). In contrast, a moderate-to-large effect size was observed for childbirth attitudes (CAQ: d = 0.70), with lower scores in parous women, and medium to large effect sizes were found for cognitive flexibility (CFI Control: d = 0.48; CFI Alternatives: d = 0.94; CFI total: d = 0.92), indicating higher cognitive flexibility in parous women.

The scores obtained from the MSPSS, CFI, CAQ, and their subdimensions were compared across sociodemographic characteristics using an independent samples t-test for normally distributed variables, whereas the Mann–Whitney U test was applied to the non-normally distributed MSPSS-Family subdimension. For comparisons involving three or more independent groups, ANOVA was conducted for variables meeting the assumption of normality, including MSPSS-Friend, Significant-Other, total MSPSS, CAQ, CFI-Control, CFI-Alternatives, and total CFI. For comparisons based on education level, where the normality assumption was violated, the non-parametric Kruskal–Wallis test was employed. In cases where a significant difference was detected, Bonferroni post-hoc tests were conducted to determine between-group differences. Significant differences in the MSPSS (*p* = 0.001), CAQ (*p* = 0.013), and CFI Control (*p* = 0.024) scores were observed across education levels. Bonferroni analysis indicated that participants with a university education had higher MSPSS, CAQ, and CFI Control scores compared to other education groups. A difference in MSPSS-Significant Other scores was found according to family type (*p* = 0.010), with higher scores in the nuclear family group compared to the extended family group. Regarding pregnancy type, significant differences were observed in MSPSS and its subdimensions (*p* < 0.05), with higher scores in participants with normal pregnancies compared to those with IVF pregnancies. No significant differences were found in total MSPSS and its subdimensions according to economic status, number of births, previous birth type, abortion/stillbirth history, or information source (*p* > 0.05). However, significant differences were observed in CAQ, CFI, and their subdimensions according to previous birth type (*p* < 0.05). Bonferroni analysis showed that participants who had a cesarean birth had lower CAQ scores than those who had vaginal birth or no previous birth.

For the CFI-Control, CFI-Alternatives, and total CFI scales, Bonferroni analysis showed that participants who never gave birth scored lower than those who had a vaginal or caesarean birth. In addition, there was a significant difference in CAQ scores based on abortion/stillbirth history (*p* = 0.008). Participants who did not have an abortion or stillbirth scored higher than those who did. There are no significant differences in CFI-Control, CFI-Alternatives, and total CFI scores (*p* > 0.05). There was a significant difference in CAQ scores based on information sources (*p* = 0.002). According to the Bonferroni analysis, participants who received information from relatives or friends and social media/TV scored significantly higher on the CAQ scale compared to those who received information from doctors. No significant differences were found in the CFI-Control, CFI-Alternatives, and total CFI scores based on information sources (*p* > 0.05).

**Table 3:** MSPSS, CFI, and CAQ scores in parous and nulliparous women

	Parous		Nulliparous			
	M. (Min.-Max.)	Mean ± Sd	M. (Min.-Max)	Mean ± Sd	Test statistic (*p*)	Effect Size
MSPSS-Family	28 (4–28)	25.35 ± 4.94	28 (8–28)	26.06 ± 3.59	Z=-0.678 (0.498)	*r* = -0.04
MSPSS-Friend	26 (4–28)	23.15 ± 5.93	26 (4–28)	23.38 ± 5.71	t=-0.364 (0.716)	d = -0.05
MSPSS-Significant other	24 (4–28)	21.23 ± 7.33	26 (4–28)	22.57 ± 6.61	t=-1.800 (0.073)	d = -0.26
MSPSS	74 (12–84)	69.74 ± 15.1	76 (28–84)	72 ± 13.16	t=-1.494 (0.136)	d = -0.21
CAQ	28 (16–61)	30.1 ± 9.85	35 (16–58)	35.28 ± 10.0	t=-4.936 (< 0.001*)	d = -0.70
CFI Control	24 (11–35)	24.44 ± 4.96	22 (11–35)	22.59 ± 5.39	t = 3.397 (< 0.001*)	d = 0.48
CFI Alternatives	52 (13–65)	50.8 ± 7.99	46 (13–65)	43.93 ± 11.5	t = 6.675 (< 0.001*)	d = 0.94
CFI	75 (44–98)	75.24 ± 9.86	69 (40–98)	66.52 ± 15.4	t = 6.529 (< 0.001*)	d = 0.92

*p < 0.05, ***p < 0.001, M: Median, Z: Mann Whitney U test statistic, t: Independent t test statistic

### Hierarchical regression for predicting CAQ

Hierarchical regression analysis was conducted to examine the incremental contribution of different variables to the prediction of childbirth attitudes. In this study, the first model included the subdimensions of the social support scale (MSPSS) to evaluate their effect on CAQ. In the second model, the subdimensions of the Cognitive Flexibility Inventory (CFI) were added to determine their additional contribution to the model. In Model 1, which includes the social support subscales (MSPSS-Family, MSPSS-Friend, and MSPSS-Significant Other), the model explained only 2% of the variance in CAQ scores (R² = 0.020), with an adjusted R² of 0.012, indicating a minimal effect size. The change in R² was not statistically significant (*p* = 0.065), suggesting that perceived social support is not significantly associated with childbirth attitudes. Table 4 shows the contribution of each variable.

**Table 4:** Hierarchical regression analysis predicting CAQ (*N* = 362)

					95% Confidence Interval for B		
	B	Std. Error	t	*p*	Lower Bound	Upper Bound	VIF
*Model 1*							
(Constant)	35.969	3.192	11.27	0.000*	29.693	42.246	
MSPSS-Family	0.146	0.157	0.931	0.352	-0.163	0.455	1.658
MSPSS-Friend	-0.232	0.124	-1.862	0.063	-0.476	0.013	1.827
MSPSS-Significant other	-0.089	0.092	-0.967	0.334	-0.269	0.092	1.449
*R²* *ΔR²* *F* *p*	0.020 0.020 2.428 0.065						
*Model 2*							
(Constant)	40.04	3.957	10.119	0.000*	32.258	47.821	
MSPSS-Family	0.174	0.154	1.133	0.258	-0.128	0.477	1.665
MSPSS-Friend	-0.228	0.122	-1.866	0.063	-0.468	0.012	1.843
MSPSS-Significant other	-0.094	0.09	-1.044	0.297	-0.27	0.083	1.449
CFI Control	-0.460	0.11	-4.168	0.000*	-0.677	-0.243	1.213
CFI Alternatives	0.128	0.056	2.272	0.024*	0.017	0.238	1.221
*R²* *ΔR²* *F* *p*	0.067 0.047 8.899 0.000						

Note. B: unstandardized regression coefficient; ΔR²: change in R²; 95% confidence intervals are reported for unstandardized coefficients

*p < 0.05

In Model 2, when cognitive flexibility variables (CFI Control and CFI Alternatives) were added, the explained variance increased to 6.7% (R² = 0.067), with an adjusted R² of 0.053, indicating a small increase in the model’s explanatory power rather than a moderate improvement. The change in R² (ΔR² = 0.047) was statistically significant (*p* < 0.001), suggesting that the addition of cognitive flexibility variables improved model fit despite low overall variance explained. Specifically, CFI Control (B = -0.460, *p* < 0.001) and CFI Alternatives (B = 0.128, *p* = 0.024) significantly predicted CAQ scores, indicating that a higher ability to generate and consider alternative solutions was associated with lower childbirth anxiety and fear. However, given the relatively low R² value, the overall explanatory power of the model remained limited, accounting for only 6.7% of the variance in CAQ scores, indicating that additional factors not included in the model may contribute substantially to childbirth attitudes. The social support subscales remained non-significant in Model 2, indicating that their contribution to the prediction of childbirth attitudes was not statistically meaningful within the final model. Overall, cognitive flexibility demonstrated a statistically significant but modest predictive contribution rather than representing a primary determinant of childbirth attitudes.

To further examine whether the relationships between CFI, MSPSS, and CAQ differed between parous and nulliparous women, a moderated hierarchical regression analysis was conducted. To reduce model complexity and potential multicollinearity in the interaction terms, and to examine construct-level effects, total scores of MSPSS and CFI were used in the moderation analysis instead of their sub-dimensions. The results indicated that being parous versus nulliparous significantly moderated the relationship between CFI and CAQ (B = -0.197, *p* = 0.027), whereas no significant moderating effect was observed for MSPSS. However, the inclusion of interaction terms did not significantly improve overall model fit (ΔR² = 0.013, *p* = 0.080), indicating a limited incremental contribution of moderation effects.

### Network analyses between study variables

**Fig. 1:**
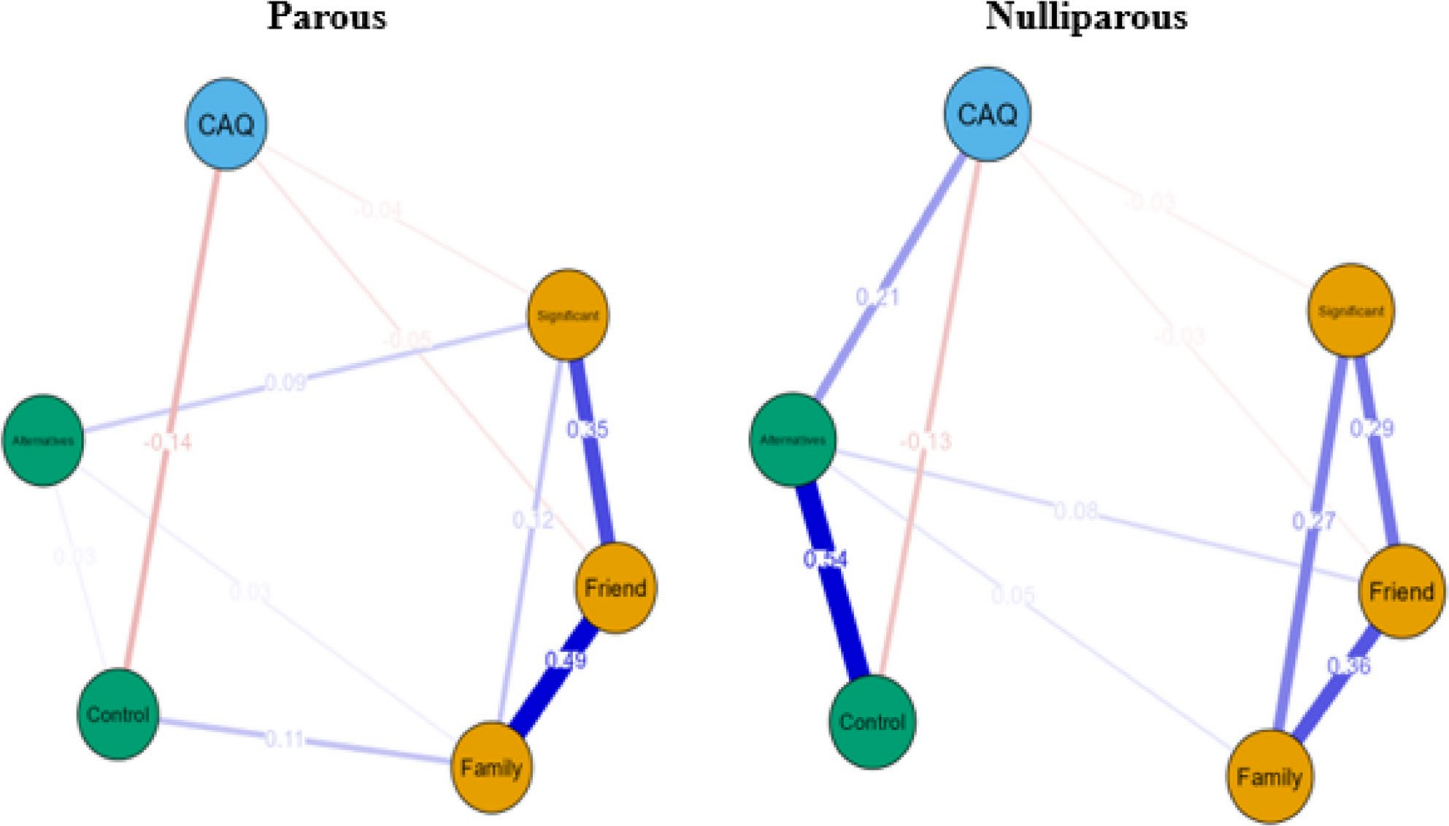
Network Plots. Note. CAQ: Childbirth Attitudes Questionnaire. Network plot illustrating the correlations among the analyzed variables/taxa. Blue and red edges indicate positive and negative correlations, respectively, while edge thickness is proportional to the strength of the correlations. Nodes represent the analyzed variables, and node labels correspond to their respective identifiers within the network

The network analysis compared associations among study variables between parous and nulliparous women and illustrated how the pattern of interconnections differs according to previous birth status. The network structure demonstrated that thick and dark blue edges, such as the association between perceived support from friends and family, represent strong positive relationships, whereas thinner and lighter edges indicate weaker associations (Fig. 1). In contrast, red edges, such as the association between control and CAQ, indicate negative relationships within the network structure. In addition, Table 5 presents centrality measures (betweenness, closeness, and strength) for each variable across parous and nulliparous groups. It should be noted that these centrality indices are standardized (z-score) measures; therefore, positive and negative values reflect relative standing of each node within the overall network structure rather than indicating the direction or valence of associations.

**Table 5:** Centrality measures per variable

Variable	Parous			Nulliparous		
	Betweenness	Closeness	Strength	Betweenness	Closeness	Strength
Alternatives	-1.220	-1.185	-1.073	1.434	1.134	1.327
CAQ	-1.220	-1.144	-0.848	-0.643	-1.437	-1.630
Control	0.244	-0.186	-0.662	-0.643	-0.049	0.032
Family	0.976	0.993	0.887	-0.643	-0.227	0.094
Friend	0.976	0.993	1.331	1.137	1.134	0.616
Significant Other	0.244	0.528	0.366	-0.643	-0.555	-0.439

In the parous group, perceived support from friends and family demonstrated the highest closeness and strength values (Friend: 1.331; Family: 0.887; closeness = 0.993 for both), indicating their prominent role in maintaining connectivity within the social support network. Accordingly, friend and family support emerged as structurally central nodes, suggesting strong interdependence among social support components in parous women.

In the nulliparous group, the strongest positive association was observed between control and alternatives, represented by the thick dark blue edge, indicating a comparatively stronger relationship than other pairwise connections within the network. The associations among perceived support from friends, family, and significant other remained relatively strong and positive, whereas the relationship between control and CAQ consistently showed a negative association, represented by red edges in the network structure.

Furthermore, in contrast to the parous group, alternatives demonstrated the highest values across all centrality indices (betweenness, closeness, and strength), suggesting that it functioned as the most central node within the nulliparous network. Overall, the network structure highlighted meaningful differences in variable interconnectedness according to previous birth status, with social support variables demonstrating greater centrality among parous women, whereas cognitive flexibility—particularly alternatives—emerged as the most central construct among nulliparous women.

## Discussion

With regard to the differences between childbirth attitudes, social support, and cognitive flexibility based on sociodemographic and obstetric factors, our results revealed the significant association of sociodemographic and obstetric factors on childbirth attitudes, perceived social support, and cognitive flexibility. In line with previous research [36, 45, 46], we found that education level was a significant factor affecting cognitive flexibility, perceived social support, and childbirth attitudes. Furthermore, the number of previous births was found to play an important role in shaping both childbirth attitudes and cognitive flexibility; however, no significant differences in perceived social support were observed based on the number of births. Furthermore, the type of previous birth, whether cesarean or vaginal, showed a significant impact on CAQ scores, with the women in our sample who had a cesarean birth scoring lower. This finding contradicts several earlier findings on the psychological effects of birth methods [47, 48]. Emergency caesarean sections are typically associated with higher postpartum anxiety, acute stress responses, and traumatic symptoms compared to vaginal births [2, 15]; however, our findings suggest a potential shift in maternal perception. In contemporary contexts, caesarean delivery may increasingly be viewed as a controllable and predictable intervention rather than solely a surgical necessity, potentially functioning as a mechanism to reduce perceived uncertainty surrounding childbirth [10]. Accordingly, for some parous women—particularly those with prior elective or well-managed caesarean experiences—the procedure may reduce subsequent childbirth fear by providing psychological predictability and a sense of control over an otherwise uncertain process [12].

Conversely, considering that cognitive flexibility was previously reported to be related to how individuals adapt to new and changing situations [20], the lack of significant differences in cognitive flexibility scores based on birth type was somewhat surprising. This outcome could be interpreted as follows: the cognitive demands and the psychological processes involved in both experiences may be similar enough that they do not result in notable differences in cognitive flexibility. Said differently, while births differ in method, the overall psychological experience of managing childbirth—be it through a cesarean or vaginal delivery—may activate similar cognitive strategies for adaptation, resulting thus in the lack of significant differences in cognitive flexibility.

Another finding that did not align with our expectations was that the women with no history of abortion or stillbirth exhibited higher CAQ scores. We anticipated that women with a history of abortion or stillbirth would experience greater fear of childbirth due to the emotional and psychological impact of such events [37, 49, 50]. Women with a history of abortion or stillbirth may have developed coping mechanisms over time, which may help reduce their fear of future childbirth. In addition, those without such experiences might have more uncertainty or anxiety about the unknown aspects of childbirth, leading to higher fear levels [11]. The higher CAQ scores observed among women without a history of abortion or stillbirth may also be understood in relation to the significantly different obstetric and socio-demographic profiles between parous and nulliparous groups. In particular, nulliparous women in the sample had higher proportions of university education and IVF pregnancies, both of which may be associated with increased awareness, planning, and consequently heightened anticipatory anxiety regarding childbirth. Moreover, differences in family structure and information sources suggest potential variability in social and informational support environments, which may further contribute to differential levels of childbirth-related fear. Significant differences in CAQ scores were found based on information sources, with the participants who relied on relatives, friends, or media/TV for childbirth information scoring higher than the women who primarily received information from doctors, which may indicate an association between informal, non-medical sources and childbirth attitudes.

Furthermore, with respect to the differences between childbirth attitudes, perceived social support, and cognitive flexibility between parous and nulliparous women, we found that, while the mean score CAQ for the total sample in was 32.40, the mean scores were 30.10 for parous women and 35.28 for nulliparous women. Consistently with previous studies conducted among diverse ethnic groups (American: 31.70, Lowe [35]; Chinese: 31.30, Qiu et al. [36]; Egyptian: 38.40, Elsharkawy et al. [51]; Greek: 31.22, Gourounti et al. [52]), our findings highlight that the absence of prior childbirth experience contributes to heightened fear of childbirth.

We found no statistically significant difference between parous and nulliparous pregnant women with regard to the perceived social support from family, friends, and significant others. These results suggest that prior childbirth experience is not significantly associated with the perceived availability or quality of social support during pregnancy. By contrast, previous studies demonstrated that factors such as the quality of relationships and the nature of individual support networks tend to play a significant role in shaping childbirth attitudes [45, 53]. This discrepancy may be attributed to the unique social context of the present study. Turkish culture is characterized by its collectivistic nature, where social ties hold significant importance [54]. From a cultural perspective, it is essential to recognize that the perception of social support among participants could remain stable over time. Emotional and practical support from close social networks may serve as a consistent and influential factor throughout pregnancy, regardless of prior childbirth experience. Therefore, while previous studies highlighted the impact of relationship quality and support networks, the stability of social support in a collectivistic culture may offer a different perspective.

Our results also indicated that cognitive flexibility and its dimensions—namely, alternatives and control—were significantly greater in parous women as compared to nulliparous women. This finding suggests that women with prior childbirth experience may possess a higher degree of cognitive flexibility when navigating the challenges associated with pregnancy. The dimension of alternatives, which refers to the ability to consider and explore different options or approaches in the event of a problem [41], was found to be greater in parous women. This could reflect a greater sense of adaptability based on their previous experiences, as they may be more accustomed to adjusting their expectations and strategies in response to the demands of pregnancy and childbirth. The experiences gained from a prior pregnancy likely provide women with a broader range of coping mechanisms and alternative solutions, thereby enhancing their cognitive flexibility [51]. Similarly, the dimension of control, which involves the ability to maintain a sense of agency and influence over one’s circumstances [41, 42], was also higher in parous women. This suggests that having prior experience with childbirth may instill in women a greater sense of confidence and mastery, enabling them to feel more in control of their pregnancy journey. The familiarity with the process likely leads to reduced uncertainty and greater perceived control over pregnancy’s physical and emotional aspects [25].

The network analysis was used as a complementary approach to the traditional analyses to examine the system-level interrelations among cognitive flexibility (control and alternatives), perceived social support (family, friends, and significant others), and CAQ in parous and nulliparous women. Unlike variable-centered methods that focus on isolated associations, this approach provides a broader view of how variables are embedded within an interconnected structure. In this sense, it offers additional insight by highlighting relatively more central variables within the network, which may represent key points of interconnectedness among cognitive flexibility (control and alternatives), perceived social support (family, friends, and significant others), and childbirth attitudes (CAQ), rather than implying causal or directional effects. In our study, this allowed for a more integrated understanding of how childbirth-related attitudes are situated within a broader network of cognitive and social factors, and how the overall pattern of interconnections differs according to previous birth experience.

Finally, as concerns perceived social support and cognitive flexibility as predictors of childbirth attitudes, the results of our hierarchical regression analyses supported the viability of cognitive flexibility and, to a lesser degree, perceived social support, in being associated with fear and attitudes about childbirth. Specifically, the inclusion of perceived support from family, friends, and significant others did not enhance the model predicting fear of childbirth, whereas the addition of cognitive flexibility dimensions—alternatives and control—made a significant contribution to the model in terms of explained variance rather than implying direct predictive power. To the best of our knowledge, our study is the first to concurrently test and compare fear of childbirth, perceived support, and cognitive flexibility.

It should be noted that our findings differ from previous studies reporting a predictive role of perceived social support in childbirth fear and attitudes [55–57]. In the present study, perceived support from family, friends, and significant others was not significantly associated with childbirth attitudes among either parous or nulliparous Turkish pregnant women. This discrepancy should be interpreted cautiously, as the current study did not directly assess culturally specific family dynamics or qualitative dimensions of social support.

One possible interpretation may relate to contextual differences in how social support is experienced within the sociocultural setting of Türkiye. In particular, family relationships, especially within extended family systems, may involve both supportive and directive components [58]. In the context of pregnancy and childbirth, such involvement may extend beyond emotional support to include advice, expectations, and participation in decision-related processes. These dynamics may influence how support is perceived; however, this remains speculative and cannot be confirmed based on the present data. Accordingly, the present findings should be considered within the broader sociocultural structure of Türkiye, where variability in family systems and differences in access to healthcare services across urban and rural settings may influence the perception and interpretation of social support during pregnancy.

Taken together, these findings suggest that the relationship between perceived social support and childbirth attitudes may be more context-dependent than previously assumed. However, this interpretation should be viewed as preliminary. Future research incorporating qualitative methodologies and culturally sensitive instruments is needed to better elucidate the mechanisms through which social support operates in shaping childbirth attitudes in Turkish populations.

The subdimensions of cognitive flexibility—alternatives and control—were more pronounced than perceived social support, potentially contributing to variations in the experience of childbirth-related fear. Indeed, women with higher flexibility in alternative dimension may be more adept at considering various ways to cope with labor, pain, and the unknown aspects of the birth process. This ability to mentally rehearse different scenarios or solutions might reduce the intensity of fear, as these women feel more equipped to handle uncertainties and potential challenges [24]. In addition, women who feel a greater sense of control over the process—be it through prior knowledge, preparation, or experience—might be less likely to experience lower levels of fear.

An unexpected finding was the positive association between CFI Alternatives and CAQ scores in the regression model. Although cognitive flexibility is generally considered protective, this result may reflect that generating multiple alternatives under uncertainty could also be associated with increased cognitive burden or worry in the context of childbirth. Alternatively, this pattern may be influenced by suppression effects within the model or the conceptual distinction between the Control and Alternatives subdimensions. Taken together, our results indicate that cognitive flexibility plays a vital role in childbirth-related fear among both parous and nulliparous women.

Extending these findings, the present results further suggest that parity (parous versus nulliparous status) may influence how cognitive flexibility relates to childbirth attitudes. Specifically, the relationship between cognitive flexibility and childbirth-related fear appears to differ depending on prior childbirth experience, indicating that cognitive processes may not function uniformly across women with different reproductive histories. One possible explanation is that prior childbirth experience may reshape how cognitive strategies are applied when dealing with uncertainty, thereby altering the psychological impact of cognitive flexibility on childbirth attitudes. In contrast, no moderating effect was observed for perceived social support, which may reflect the relatively stable and culturally embedded nature of social support perceptions within the study context. However, the limited improvement in overall model fit suggests that, although statistically detectable, these moderating effects contribute only modestly to the explanation of childbirth attitudes. Overall, these findings highlight that parity may subtly shape cognitive–emotional mechanisms related to childbirth fear, but its explanatory influence remains limited.

### Theoretical and practical implications

The results of this study provide important implications for both clinical practice and future research. First, the significant associations of sociodemographic and obstetric factors—such as education level, previous birth experiences, and obstetric history—on childbirth attitudes, perceived social support, and cognitive flexibility suggest that healthcare providers should consider these factors when offering support and guidance to pregnant women. For instance, our finding that education level has a strong association with more positive childbirth attitudes and greater cognitive flexibility underscores the need for structured, content-specific educational interventions, particularly for women with lower educational attainment. Such programs should go beyond general information provision and may include evidence-based childbirth education modules, cognitive flexibility training (e.g., reframing catastrophic thoughts and improving adaptive thinking strategies), and fear-of-childbirth counseling that directly targets anxiety, uncertainty, and perceived loss of control. Furthermore, the observed impact of prior birth experiences, such as the number and type of birth, on childbirth attitudes and cognitive flexibility highlights the importance of personalized antenatal care pathways. In this context, interventions may incorporate coping-skills training (e.g., breathing techniques, relaxation strategies, and pain-coping rehearsal) and guided preparation sessions that enhance perceived control over the birth process through scenario-based planning and decision-making support. Since cognitive flexibility is a modifiable psychosocial factor that contributes to childbirth-related fear, such targeted and skills-based interventions may be particularly beneficial for nulliparous women [59]. The lack of a significant effect of social support on childbirth fear, along with the higher fear associated with information from family, friends, and social media, suggests that, in Turkish culture, informal sources may contribute to increased anxiety [60]. This highlights the need for healthcare providers to offer accurate, evidence-based information and help women navigate these potentially stressful experiences more effectively.

### Limitations

The present study has several limitations. First, the cross-sectional design of the study does not allow us to draw causal conclusions. Therefore, the findings should be interpreted as associative rather than causal due to the study design. Consequently, the use of self-report measures and recruitment from a single center may have introduced self-report and selection biases. Importantly, the exclusion of women receiving psychiatric treatment may have reduced the variability in childbirth fear within the sample and may limit the applicability of the findings to psychologically more vulnerable pregnant populations. Future research employing longitudinal designs would better capture how sociodemographic and obstetric factors influence childbirth attitudes and cognitive flexibility over time, particularly in the context of prenatal care. Furthermore, although previous studies have highlighted the positive role of perceived social support in reducing childbirth fear, these associations may be more complex in collectivistic contexts such as Türkiye, where family involvement and informal support networks are highly salient. In this regard, a further limitation of the present study is that the measure of perceived social support does not capture qualitative dimensions of support (e.g., intrusive or ambivalent support), which may be particularly relevant in such cultural settings. Future research should therefore consider the multidimensional and context-dependent nature of social support when examining its relationship with childbirth-related outcomes. In addition, this study did not measure potentially relevant psychological and experiential variables such as anxiety sensitivity, trauma history, or the quality and content of childbirth education, which may also be associated with childbirth attitudes and fear and may partially explain the observed relationships reported in this study. Accordingly, the relatively low explained variance further suggests that other unmeasured variables may have played a role in the observed associations. Lastly, the absence of clinical outcome data, including actual mode of delivery, postpartum depression, birth satisfaction, and subjective birth experience, which limits the ability to directly link the observed psychological constructs with real obstetric and postnatal outcomes.

## Conclusions

The present study highlights sociodemographic and obstetric characteristics alongside cognitive flexibility as relevant factors associated with childbirth attitudes. It is important to note that social support in this study reflects perceived availability rather than its quality or potential ambivalence; therefore, the absence of a significant association should not be interpreted as indicating that social support is unimportant in this context. Cognitive flexibility emerged as a relatively more consistent correlate within the examined variables, although the observed associations were modest and do not support causal inferences. Overall, these findings suggest that cognitive flexibility may represent a potentially relevant psychological factor that warrants further investigation in longitudinal and interventional studies to clarify its role in shaping childbirth-related psychological outcomes.

## Data Availability

The datasets used and/or analyzed during the current study are available from the corresponding author upon reasonable request.

## Abbreviations

CAQ: Childbirth Attitudes Questionnaire
MSPSS: Multidimensional Scale of Perceived Social Support
CFI: Cognitive Flexibility Inventory
